# Mutable Collagenous Tissue Isolated from Echinoderms Leads to the Production of a Dermal Template That Is Biocompatible and Effective for Wound Healing in Rats

**DOI:** 10.3390/md21100506

**Published:** 2023-09-26

**Authors:** Anna Carolo, Luca Melotti, Giulia Zivelonghi, Roberta Sacchetto, Eylem Emek Akyürek, Tiziana Martinello, Andrea Venerando, Ilaria Iacopetti, Michela Sugni, Giordana Martinelli, Margherita Roncoroni, Stefania Marzorati, Silvia Barbon, Martina Contran, Damiana Incendi, Filippo Perozzo, Andrea Porzionato, Vincenzo Vindigni, Marco Patruno

**Affiliations:** 1 Department of Comparative Biomedicine and Food Science, University of Padua, 35020 Legnaro, Italy; anna.carolo@unipd.it (A.C.); giulia.zivelonghi@studenti.unipd.it (G.Z.); roberta.sacchetto@unipd.it (R.S.); eylememek.akyurek@phd.unipd.it (E.E.A.); 2 Department of Veterinary Medicine, University of Bari, 70010 Valenzano, Italy; tiziana.martinello@uniba.it; 3 Department of Agricultural, Food, Environmental and Animal Sciences, University of Udine, 33100 Udine, Italy; andrea.venerando@uniud.it; 4 Department of Animal Medicine, Production and Health, University of Padova, 35020 Legnaro, Italy; ilaria.iacopetti@unipd.it; 5 Department of Environmental Science and Policy, University of Milan, 20133 Milan, Italy; michela.sugni@unimi.it (M.S.); giordana.martinelli@unimi.it (G.M.); margherita.roncoroni@studenti.unimi.it (M.R.); stefania.marzorati@unimi.it (S.M.); 6 Department of Neuroscience, University of Padua, 35121 Padova, Italy; silvia.barbon@unipd.it (S.B.); martina.contran@unipd.it (M.C.); damiana.incendi@unipd.it (D.I.); andrea.porzionato@unipd.it (A.P.); vincenzo.vindigni@unipd.it (V.V.); 7 Plastic and Reconstructive Surgery Unit, Padova University Hospital, 35128 Padova, Italy; filippo.perozzo@gmail.com

**Keywords:** wound healing, marine collagen, mutable collagenous tissue, circular economy, blue biotechnologies, skin regeneration, biomaterial, sea urchin, marine food waste, zero-waste approach

## Abstract

The mutable collagenous tissue (MCT) of echinoderms possesses biological peculiarities that facilitate native collagen extraction and employment for biomedical applications such as regenerative purposes for the treatment of skin wounds. Strategies for skin regeneration have been developed and dermal substitutes have been used to cover the lesion to facilitate cell proliferation, although very little is known about the application of novel matrix obtained from marine collagen. From food waste we isolated eco-friendly collagen, naturally enriched with glycosaminoglycans, to produce an innovative marine-derived biomaterial assembled as a novel bi-layered skin substitute (Marine Collagen Dermal Template or MCDT). The present work carried out a preliminary experimental in vivo comparative analysis between the MCDT and Integra, one of the most widely used dermal templates for wound management, in a rat model of full-thickness skin wounds. Clinical, histological, and molecular evaluations showed that the MCDT might be a valuable tool in promoting and supporting skin wound healing: it is biocompatible, as no adverse reactions were observed, along with stimulating angiogenesis and the deposition of mature collagen. Therefore, the two dermal templates used in this study displayed similar biocompatibility and outcome with focus on full-thickness skin wounds, although a peculiar cellular behavior involving the angiogenesis process was observed for the MCDT.

## 1. Introduction

Skin is the largest organ in vertebrates, and it represents the first barrier against the external environment; it has a key role in several life-preserving processes, such as thermoregulation, modulation of water loss, protection against pathogens and chemicals, and vitamin D synthesis. A loss in its integrity results therefore in a functional imbalance that, in severe cases, can lead to death [1].

Skin wound healing is a complex process consisting of four overlapping phases (hemostasis, inflammation, proliferation, and remodeling), which are mediated by the interaction of multiple cell types and numerous cytokines, chemokines, and growth factors [2].

Moreover, the repair of skin wounds in adults commonly results in the formation of fibrotic tissue (scar); this process differs from regeneration as scars consist of disorganized extracellular matrix, whereas regeneration leads to the formation of a tissue that possesses identical structural and functional properties, almost indistinguishable from those of the original skin [1].

The gold standard for the treatment of large or severe non-healing wounds is the application of skin autografts [3]. However, the use of this practice is limited by difficulties in finding a healthy tissue donor and the intense pain it causes the patients. In addition, graft failure is frequent due to excessive inflammation, infection, or animal movement [3,4]. Therefore, in veterinary patients, large skin wounds are either treated via skin flaps or conservatively treated to heal by secondary intention.

For the above-mentioned reasons, both in human and in veterinary medicine, the demand for products that can accelerate and improve the healing process is increasing, as is research in this field [5].

Tissue engineering is offering promising solutions by creating skin substitutes that can accelerate wound closure and enhance the quality of the newformed tissue. Skin substitutes can be described as “a heterogeneous group of biological and/or synthetic elements that enable the temporary or permanent occlusion of wounds” [6]. The ideal scaffold should be resistant, easy-to-handle, and should resemble the skin structure and function as closely as possible.

Previously, materials of different origin (bovine collagen, polyglycolic acid, acellular cadaver dermis, etc.), have been utilized to obtain skin substitutes [7,8,9,10]. Their main constituent is collagen, in addition to which lipids, fibrin, glycosaminoglycans, and proteoglycans can also be present.

As skin substitutes should resemble the physiological anatomy of the skin, many of them are designed with a bi-layered structure, such as Alloderm^®^ [11,12], Integra^®^ (IDRT, Integra Dermal Regenerative Template) [13], Pelnac^®^ [14], and Matriderm^®^ [15,16]. These products are of vertebrate animal origin, which raises both economical and ethical problems because of the risk of disease transmission (such as Bovine Spongiform Encephalopathy). Alternative collagen sources have been investigated (e.g., marine organisms) [17,18,19] and echinoderms are one of the most promising of these. In fact, since hydrolysis is a step required for effective extraction, most marine-derived collagens (from fish, sponges, jellyfish, mollusks, and other organisms), as well as bovine or porcine collagens, are employed in their hydrolyzed state, though this chemical extraction method has two main drawbacks. In order to replicate the natural properties (e.g., hydration) of the extracellular matrix (ECM), collagen-associated molecules or glycosaminoglycans (GAGs) must first be artificially introduced because they are typically lost during hydrolysis [20]. Echinoderm tissues, including those of sea urchins, easily yield large amounts of collagen fibrils in their native conformation, with retained endogenous fibril-associated GAGs [21,22,23], thus avoiding the need for hydrolysis. This peculiarity is due to the intrinsic and unique features of the collagenous tissue from which echinoderm collagen is extracted, i.e., the mutable collagenous tissue (MCT). Indeed, in contrast to vertebrate connective tissue, MCT is characterized by the absence of permanent interfibrillar crosslinks [24], which facilitates their extraction while maintaining their full integrity and mechanical efficiency [21,22]. This collagen displays structural features (D-period, chain composition, etc.) comparable with those of mammals [25], therefore representing a potential alternative tool in biomedical applications. Furthermore, the recent optimization of extraction procedures enables this collagen to be obtained from a food by-product, i.e., the sea urchin peristomial membrane, a well-known mutable collagenous structure [26], which is discarded, together with the tests and the spines, once edible sea urchins are processed for their gonads [21]. This eco-friendly collagen was efficiently used to produce an innovative marine-derived biomaterial and particularly a novel bi-layered skin substitute (Marine Collagen Dermal Template or MCDT) [27,28]. This device was recently characterized in terms of ultrastructure, mechanical stability, functionality, and in vitro cytocompatibility [27]. Furthermore, a preliminary study in a large animal model showed promising results with the MCDT supporting and promoting the physiological healing of the skin, especially at the structural level [19].

As a step forward, the aims of the current study were to apply this novel skin substitute (MCDT) on in vivo experimental wounds to assess its regeneration efficacy, and to compare it with a commercial product already in use in human medicine (IDRT).

## 2. Results

### 2.1. Clinical Follow-Up

The macroscopic appearance of each lesion site was evaluated at 5 and 10 days post-surgery: no signs of fluid accumulation, adverse inflammatory reactions, or infection were noted during all studied periods.

The presence of the biomaterials Integra Dermal Regeneration Template (IDRT) and Marine Collagen Dermal Template (MCDT) was observed in treated wounds at both time-points. IDRT at day 5 presented with a yellowish appearance due to the presence of the silicone layer, while the IDRT at day 10 and MCDT at both time points appeared as tissue with a reddish appearance.

**Figure 1:**
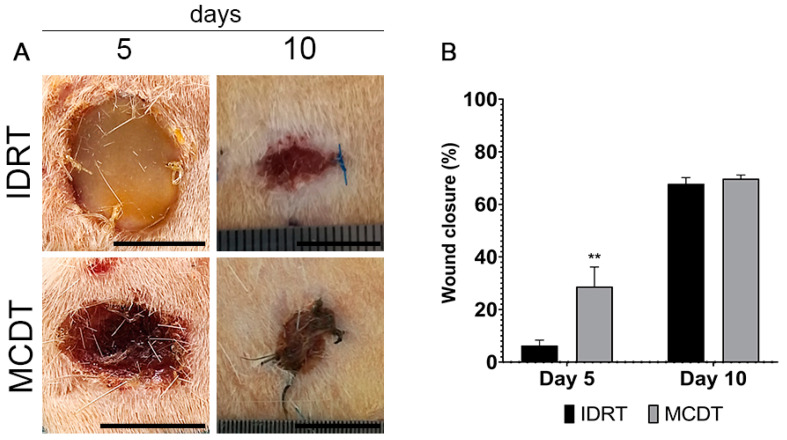
Clinical aspect of wounds. (**A**) Representative images of skin lesions at 5 and 10 days after wounding and (**B**) wound closure ratio. Data are expressed as mean ± SEM; ** *p* < 0.01 by Student’s *t*-test. Scale bar = 1 cm.

When evaluating the wound contraction percentage, 5 days after surgery, the wounds treated with MCDT showed a statistically significant acceleration of wound closure compared with wounds treated with IDRT (MCDT 28.74 ± 3.73% vs. IDRT 6.32 ± 1.44%, *p* < 0.01). At 10 days, wound closure was similar between treatment groups (MCDT 69.79 ± 0.80% vs. IDRT 67.81 ± 1.36%) (Figure 1). At 10 days, none of the treatment groups reached a complete wound closure.

### 2.2. Histological Analysis

**Figure 2:**
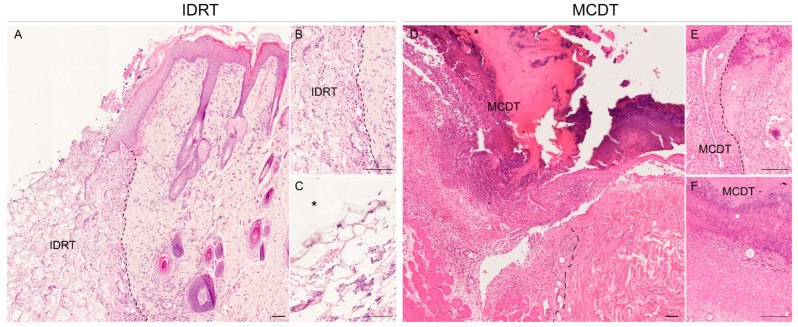
Histological microphotographs of skin biopsies at 5 days after wounding: comparison of IDRT- (**A**–**C**) and MCDT-treated (**D**–**F**) wounds. (**A**) Skin wounds treated with Integra (IDRT) showed an abundant inflammatory infiltrate in different regions of the dermis and a silicon layer on top. (**B**) Higher magnification detail of wound border into contact with the dermal template (separated by the dotted line. (**C**) Higher magnification detail of cell infiltration in the dermal template. (**D**) Skin wounds treated with MCDT showed similar features. (**E**) Higher magnification details of the wound border and (**F**) of cell infiltration in the dermal template. Abbreviations: IDRT = Integra Dermal Regeneration Template, * = silicone layer of IDRT, MCDT = Marine Collagen Dermal Template. Scale bar = 200 µm.

**Figure 3:**
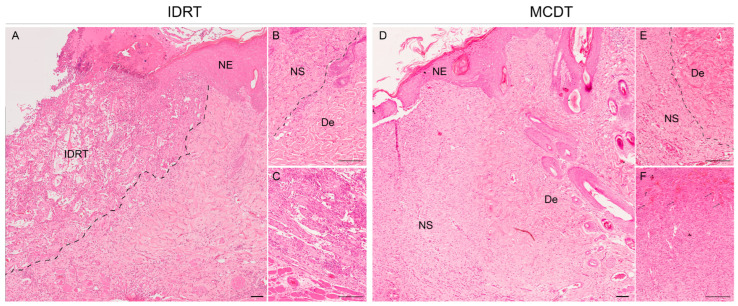
Histological microphotographs of skin biopsies at 10 days after wounding: comparison of IDRT- and MCDT-treated wounds. (**A**) Skin wounds treated with IDRT showed an abundant cell infiltrate and a neo-epidermis (NE) in the wound border. (**B**) Higher magnification detail of the border with the newly formed skin (NS), developed from the granulation tissue, and the wound border, mature dermis (De). (**C**) Neovascularization in IDRT-treated wounds in the lower part of the dermis. (**D**) Skin wounds treated with MCDT showed cell infiltration in different areas of the dermis along with a neo-epidermis (NE). (**E**) Higher magnification detail of the border between the newly developed dermis (NS) and the mature dermis from the unwounded skin (De). (**F**) Presence of vessels in the dermal layer in the central area of the wound. Abbreviations: IDRT = Integra Dermal Regeneration Template, NE = Neoepidermis, NS = Neoskin, De = mature dermis. Scale bar = 200 µm.

Histological observations of wounds treated with MCDT and IDRT led to similar results. At 5 days wounds covered by IDRT showed the “silicone” layer at the top and a “matrix web” underneath, in the deep region of the dermis; the latter region showed an abundant inflammatory infiltrate (Figure 2A) while a mild inflammation was observed in superficial areas (Figure 2B,C). At the same stage, wounds covered by MCDT showed a copious inflammatory infiltrate deep in the dermis (Figure 2D) and a minor infiltrate close to the surface (Figure 2E,F). The deposition of granulation tissue was similar between the two treatments, with the difference that in MCDT-treated lesions a major presence of granulation tissue beneath the skin substitute was noted rather than cellular infiltration in the scaffold as observed in IDRT-treated wounds; indeed, in the latter the granulation tissue was absent in 50% of wounds while 50% showed a mild presence of it. On the other hand, 50% of MCDT-treated wounds showed a moderate presence of granulation and the remaining 50% an abundant one. At 10 days, wounds covered by IDRT and MCDT showed a similar pattern (Figure 3), although processes of angiogenesis were more extensive in the MCDT-treated wounds (Figure 3F). The lesions treated with IDRT showed a higher presence of immature granulation tissue (Figure 3A,B) (50% mild, 25% moderate, and 25% abundant) compared to MCDT-treated lesions (Figure 3D,E) (25% absent and 75% mild presence); furthermore, the granulation tissue in these lesions presented with a higher grade of maturation compared to wounds treated with IDRT.

### 2.3. Gene Expression Analysis

**Figure 4:**
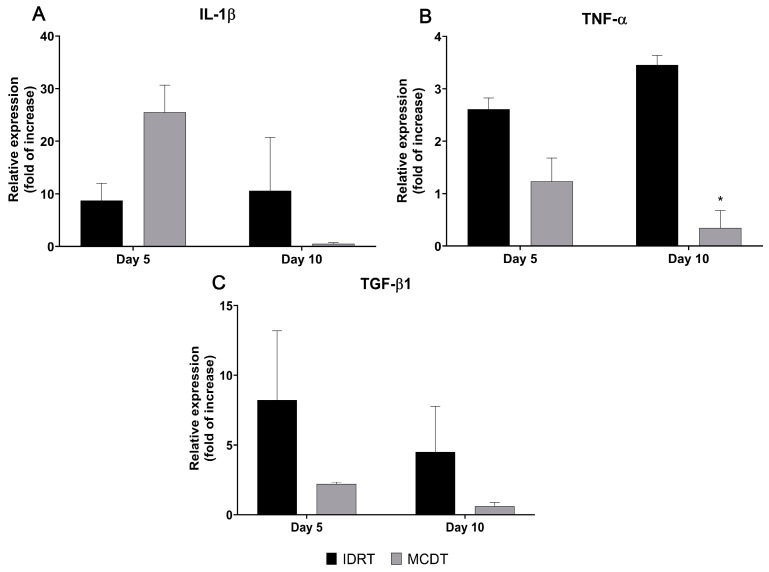
Gene expression of pro-inflammatory cytokines involved in the wound healing process. The mRNA levels of (**A**) interleukin-1 beta (IL-1β), (**B**) tumor necrosis factor alpha (TNF-α), and (**C**) transforming growth factor beta 1 (TGF-β1) were assessed in both experimental groups (IDRT and MCDT) at 5 and 10 days after wounding and compared with each other. Relative gene expression levels were normalized using two reference genes (GAPDH and β-actin) and uninjured skin was used as the calibrator sample. * *p* < 0.05 by Student’s *t*-test.

In RT-PCR analysis of IL-1β gene expression, the MCDT-treated group at 5 days showed a relative gene expression almost three times higher than that of the IDRT-treated group (MCDT 25.48 ± 5.18 vs. IDRT 8.75 ± 3.26). An opposite trend of expression could be noted at day 10, where IL-1β gene expression was almost 20-fold lower in the MCDT- than in the IDRT-treated group (MCDT 0.49 ± 0.22 vs. IDRT 10.59 ± 10.16) (Figure 4A).

TNF-α gene expression at day 5 was lower in the MCDT-treated group than in the IDRT-treated group (MCDT 1.23 ± 0.44 vs. IDRT 2.61 ± 0.22); at 10 days the difference between treatment groups was statistically significant, with a gene expression 10 times lower in the MCDT- than in the IDRT-treated group (MCDT 0.34 ± 0.33 vs. IDRT 3.46 ± 0.18, *p* = 0.014) (Figure 4B).

TGF-β1 gene expression at day 5 was three times lower in the MCDT- than in the IDRT-treated group (MCDT 2.19 ± 0.14 vs. IDRT 8.21 ± 4.96). At 10 days, a decrease in TGF-β1 gene expression could be noted in both groups, with the MCDT group relative expression 7-fold lower than the IDRT-treated group relative expression (MCDT 0.61 ± 0.27 vs. IDRT 4.49 ± 3.29) (Figure 4C).

**Figure 5:**
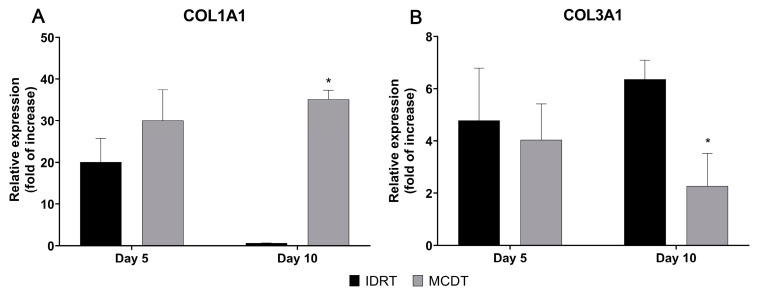
Gene expression analysis of collagen type I and III, which are involved in the wound healing process. The mRNA levels of (**A**) collagen type IA1 (COL1A1) and (**B**) collagen type IIIA1 (COL3A1) were assessed at 5 and 10 days after wounding in IDRT- and MCDT-treated wounds. Relative gene expression levels were normalized using two reference genes (GAPDH and β-actin) and uninjured skin was used as the calibrator sample. * *p* < 0.05 by Student’s *t*-test.

Taking into consideration COL1A1, RT-PCR revealed an up-regulation of the relative expression in the MCDT-treated group when compared with the IDRT treatment group. This was particularly notable and statistically significant at 10 days, where the MCDT group had a 50-fold higher relative expression of COL1A1 than the IDRT treatment group (MCDT 35.16 ± 2.14 vs. IDRT 0.65 ± 0.02, *p* = 0.04). The difference in COL1A1 gene expression between MCDT and IDRT treatment groups at 5 days was less evident and not statistically significant (MCDT 29.99 ± 7.44 vs. IDRT 20.07 ± 5.67) (Figure 5A).

The expression of COL3A1 was instead higher in the IDRT than in the MCDT treatment group at both time points, with a lower and statistically significant difference at day 10 compared to IDRT (day 5: MCDT 4.04 ± 1.38 vs. IDRT 4.78 ± 2.00; day 10: MCDT 2.27 ± 1.25 vs. IDRT 6.36 ± 0.73, *p* = 0.048) (Figure 5B).

**Figure 6:**
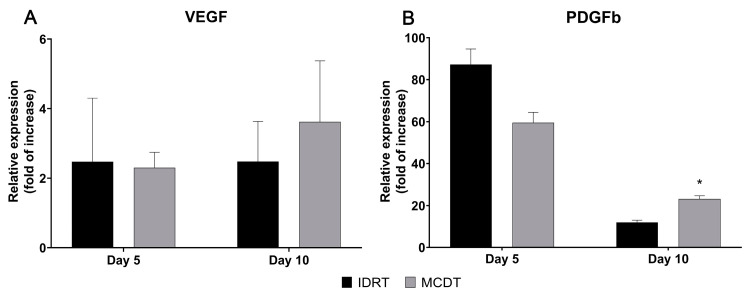
Gene expression analysis of growth factors promoting angiogenesis during wound healing. The mRNA levels of (**A**) vascular endothelial growth factor (VEGF) and (**B**) platelet-derived growth factor B (PDGFb) were assessed at 5 and 10 days after wounding in IDRT- and MCDT-treated wounds. Relative gene expression levels were normalized using two reference genes (GAPDH and β-actin) and uninjured skin was used as the calibrator sample. * *p* < 0.05 by Student’s *t*-test.

At day 5, PDGFb gene expression was lower in the MCDT than in the IDRT treatment group (MCDT 59.57 ± 4.88 vs. IDRT 87.32 ± 7.37), while VEGF gene expression was comparable (MCDT 2.30 ± 0.44 vs. IDRT 2.47 ± 1.83) (Figure 6).

At 10 days the expression was higher in the MCDT treatment group for both PDGFb (MCDT 23.18 ± 1.47 vs. IDRT 11.89 ± 1.09, *p* = 0.025) and VEGF (MCDT 3.62 ± 1.75 vs. IDRT 2.48 ± 1.16) gene expression (Figure 6).

## 3. Discussion

The mutable collagenous tissue (MCT) of echinoderms shows biological peculiarities that facilitate extraction of native collagen and its subsequent use for regenerative medicine purposes, including skin wound treatment [19,27]. The relative ease of obtaining collagen fibrils in their native conformation (as they are in the ECM) enables the production of highly biomimetic biomaterials of marine origin, which are structurally and biochemically similar to the dermis [22,27], and therefore can potentially promote skin regeneration processes [19].

Skin wound healing is a crucial process, and its dysregulation may result in conditions such as pathological scarring or non-healing wounds, which might greatly affect the quality of life [1,3,29]. For this reason, attempts to find novel therapies or solutions to facilitate skin regeneration are crucial from a socio-economic point of view. In recent years, in order to accelerate and improve wound healing, different skin substitutes have been designed [28,30,31] that are mainly based on the most abundant components of the ECM [32,33,34], such as collagen [7,8,9]. This latter is mainly of mammalian origin, a feature that may present medical and ethical concerns; therefore, an economically sustainable and disease-transmission safe collagen source is still needed and marine collagens are a promising alternative [35]. In line with this challenge, the collagen used in the present work was obtained from seafood industry by-products and particularly from a specific mutable collagenous structure (the peristomial membrane) derived from discarded waste of the purple sea urchin (*Paracentrotus lividus*). The cytocompatibility of this structure has previously been tested in in vitro [21,22,28] and partially in in vivo [19] studies, where a marine collagen biomaterial promoted wound healing in sheep skin experimental wounds.

The aim of this preliminary study was to compare this innovative biomaterial, herein referred to as Marine Collagen Dermal Template (MCDT), with Integra Dermal Regenerative Template (IDRT) and evaluate its regenerative efficacy and biocompatibility on experimental skin wounds induced in an in vivo rat model.

After skin injury, the damaged cells and the exposed tissues activate platelet aggregation with subsequent clot formation and release of chemotactic factors leading to the inflammatory phase of wound healing [36,37,38]. Under normal circumstances, inflammation is a self-limiting process that allows the progression of healing by removing necrotic tissue, debris, and pathogens. In some pathological conditions though, inflammation persists, slowing down the healing process and leading to wound chronicity [39,40].

In the current study, inflammation was evaluated macroscopically and histologically as well by gene expression analysis of pro-inflammatory factors. While no clinical signs of inflammation were noted (redness, swelling, and movement impairment), histological analyses showed that at 5 days after treatment wounds treated with MCDT displayed a higher inflammatory infiltrate compared with wounds treated with IDRT. At 10 days, the inflammatory infiltrate was similar between the two treatments. The higher inflammatory reaction noted in the early phase in the MCDT-treated group is probably correlated with the mRNA levels of IL-1β and TNF-α, both well-studied pro-inflammatory cytokines [41]. Indeed, IL-1β relative gene expression at day 5 was almost three times higher in the MCDT than in the IDRT treatment group, whereas an opposite trend of expression could be noted at day 10, where IL-1β gene expression was almost 20-fold lower in the MCDT than in the IDRT treatment group. Moreover, TNF-α gene expression in the MCDT treatment group decreased between day 5 and day 10, reaching at day 10 a relative gene expression 10-fold lower than the one observed in IDRT treatment group.

Histological results are consistent with previous observations on the sheep animal model [19] where the inflammatory infiltrate was higher during the first week but diminished throughout the experimental period and may therefore confirm that our biomaterial anticipates the activation of the inflammatory phase by an earlier activation of platelets. In fact, collagen in its native fibrillar conformation, like the MCT-derived collagen used in the MCDT, can activate platelets inducing their degranulation [42,43] and therefore the release of several soluble factors, including chemotactic molecules for inflammatory cells [37,44,45,46]. It is possible that the earlier activation of platelets might accelerate the first phases of wound healing and subsequently shortening of the inflammatory phase, anticipating the transition to the subsequent stage of healing.

During the final stages of inflammation, the proliferation phase begins. This includes the accumulation of immature extracellular matrix and the formation of the granulation tissue that is particularly important in wounds that heal by secondary intention, as in our experimental model, since it serves as scaffold for other cells along with blood vessels and sustains wound contraction [36,37].

The biocompatibility and ability to support cellular infiltration and proliferation of our biomaterial was previously demonstrated in vitro and in a large size animal model (sheep) in vivo; indeed, the 3D sponge-like structure of MCDT supported cellular migration, proliferation in vitro [27], and the deposition of granulation tissue in skin wound lesions in the sheep model [19]. At the histological level, wounds presented with a different amount of granulation tissue. Lesions treated with MCDT showed an accelerated and more abundant deposition of granulation tissue at day 5 compared to wounds treated with IDRT. On the contrary, at day 10 an opposite trend was observed with a higher presence of immature granulation tissue in IDRT-treated wounds; however, the lower presence of granulation tissue in MCDT-treated wounds might be ascribed to the fact that the granulation tissue is currently developing to a mature dermis, hence the lesser amount observed.

In order to reach a proper stage of maturation, granulation tissue needs an adequate blood supply provided by endothelial cells; the latter cells need to grow quicky in the newly formed tissue to restore the flow of oxygen and nutrients to the site [47,48]. Several angiogenic factors, such as vascular endothelial growth factor (VEGF) and platelet-derived growth factor (PDGF), are involved in triggering the activation of the local endothelial cells [37,48,49]. In the present research, the relative gene expression of these two growth factors was evaluated. At 5 days post-injury, the relative expression of both pro-angiogenic factors was comparable between treatment groups; in contrast, at 10 days post-injury both VEGF and PDGFb relative expression was higher in MCDT-treated wounds. The high levels of expression observed for these two growth factors at 10 days might have increased the angiogenic process in MCDT-treated wounds, promoting the maturation of granulation tissue, thus supporting wound healing [49,50,51]. Similarly, from a histological perspective, angiogenesis appeared to be more diffuse in MCDT-treated wounds at 10 days post-injury compared to IDRT-treated wounds evaluated at 10 days post-injury.

In physiological conditions, as the wound heals, the borders of the lesion are typically pulled towards the center of the wound, reducing the size of the open wound area [52]. This process, called wound contraction, is particularly important in rodent skin wound healing, as these animals possess a thick *Panniculus carnosus* under the dermis layer [53,54,55].

Regarding the contraction rate in the present study, wounds treated with MCDT showed a statistically significant greater degree of wound closure compared with wounds treated with IDRT: the wound contraction percentage at 5 days post-injury was four-fold higher in MCDT- than in IDRT-treated wounds. On the other hand, at 10 days none of the experimental groups reached a complete wound closure, and wound contraction percentage was comparable between treatments. These results might be due to earlier activation of the inflammatory phase and higher vascular infiltration of the granulation tissue that have anticipated the wound contraction in MCDT-treated wounds [50].

The last phase of wound healing is remodeling, which consists of regression of the newly formed vessels and replacement of immature ECM components [34], a process that can be assessed by analyzing the type of collagen expressed during wound healing. As a matter of fact, granulation tissue is largely composed of collagen type III, which is gradually replaced by collagen type I as the maturation of the wounded tissue progresses [56,57].

In MCDT-treated wounds, the gene expression of collagen type III was lower than in IDRT-treated wounds both at 5 and 10 days post treatment; gene expression of mature collagen (collagen I) was instead higher at both time points, reaching higher values than in the IDRT treatment group, at day 10. The increased presence of mature collagen observed in MCDT-treated wounds might suggest that the marine collagen biomaterial has promoted the physiological maturation of the skin lesion.

Furthermore, the difference in cell infiltration between the two templates suggests that MCDT is not completely integrated into the healing tissue, in contrast to IDRT. Ongoing studies are evaluating if this could be the reason for the positive aspects shown in the present research; a larger number of animals is, however, needed to confirm the observed promising results.

The main drawback of this study is the low robustness of the obtained data due to the reduced number of animals involved in the experimentation; for this reason, this study has to be considered as a preliminary study. Despite this limitation, these promising exploratory results might be the basis for future studies involving the application of marine collagen-based biomaterials for the treatment of skin wounds.

## 4. Materials and Methods

### 4.1. Animal Model and Ethical Statement

Sixteen adult male rats (more than eight weeks old; Charles River Laboratories Italia S.R.L., Lecco, Italy) with an average weight of 360 g, were included in this experimental study and were divided into 8 animals per treatment group (IDRT or MCDT). Each treatment group (n = 8) was further divided into two even groups (n = 4) according to the time that passed after treatment (5 and 10 days). Rats were allocated to separate cages in an animal facility (DNS Department, University of Padova, Padova, Italy) with ad libitum access to food and water, and daily monitoring for signs of discomfort. Animals were housed at least two weeks prior to the start of the experimental study for acclimation.

At the end of the experiment, rats were humanely euthanized by CO_2_ overdose. The experiment was approved by the Italian Ministry of Health (n°57/2022-PR), in accordance with the Body for the Protection of Animals (OPBA).

### 4.2. Sea Urchin Collagen Extraction, Production of 3D Scaffolds

Native and GAG-decorated fibrillar collagen was extracted from the purple sea urchin (*Paracentrotus lividus*), specifically from the peristomial membranes obtained from restaurant waste, as previously described [21,22]. Briefly, the peristomial membranes (i.e., the soft membrane surrounding the mouth) were isolated from the waste, rinsed in artificial sea water, weighed, and left overnight at 23 °C in a hypotonic buffer (10 mM Tris-HCl, 0.1% EDTA, pH 8.0). After several washes in phosphate buffer saline (PBS), samples were left overnight at 23 °C in decellularizing solution (10 mM Tris-HCl, 0.1% Sodium Dodecyl Sulphate, pH 8.0). Numerous washes in PBS were performed to remove the Sodium Dodecyl Sulphate and a disaggregating solution (0.5 M NaCl, 0.1 M Tris-HCl pH 8.0, 0.1 M β-mercaptoethanol, 0.05 M EDTA) was added and left at RT for at least 5 days. All these steps were performed in stirring conditions. The resulting collagen suspension was filtered on steel mesh filter and dialyzed at RT against 0.5 M EDTA for 4 h and against distilled water overnight to completely remove the β-mercaptoethanol.

The resulting suspension of fibrillar collagen in autoclaved filtered distilled water (dH_2_O) was stored at −80 °C until use. After thawing at room temperature (RT), it was used to produce both 2D thin membranes and 3D sponge-like scaffolds following two different combined procedures, as previously described [22,27]. Briefly, 2D membranes were produced by adding 0.6 mL of collagen suspension (2 mg/mL in 0.01% TritonX-100 in autoclaved filtered dH_2_O) to rubber silicone molds (diameter 10 mm, height 10 mm) and left to dry at 37 °C overnight. The 3D scaffolds were produced by adding 1 mL of collagen suspension (6 mg/mL) in 6% ethanol in autoclaved filtered dH2O to the 2D membranes rubber silicone molds. Both 2D membranes and 3D scaffolds were placed under vacuum for 2 h to remove air bubbles, frozen overnight at −80 °C, and lyophilized (Edwards Pirani 1001) overnight. Finally, the resulting Marine Collagen Dermal Templates (MCDTs) were sterilized under a 15 W UV lamp at RT overnight and stored at −20 °C until use.

Immediately before the surgical operation, they were sterilized for 1 h under an UV lamp.

### 4.3. Surgical Procedure and Clinical Follow-Up

General anesthesia was induced delivering 4.5% isoflurane/oxygen-medical air in an induction chamber, and then maintained with a facial mask at 1–1.5% isoflurane/oxygen-medical air. After anesthesia induction, the animals were placed in sternal recumbency, and the trichotomy of the back was performed. The surgical field was scrubbed with 10% iodine-povidone and the wound areas were marked using a sterilized guide to ensure the correct diameter and distance of each lesion. The skin was then incised with a surgical blade and removed using dissection scissors creating two full thickness round skin lesions with a diameter of 1 cm symmetrically lateral of the dorsal column with a distance between them of 1.5 cm, ensuring that the healing process of one lesion would not affect the other.

On each rat, one lesion was treated with either IDRT or MCDT while the second one was left untreated and considered a control lesion. IDRT or MCDT was applied and securely fixed to the lesion site with surgical stitches. Tegaderm^TM^+Pad plaster was applied on top of the lesion site and secured with surgical stitches.

The animals received analgesic and antibiotic therapy for four days after surgery (Carprofen 10 mg/kg SC, Enrofloxacin 5 mg/kg SC) and were daily monitored for signs of discomfort; no clinical signs of infection or excessive inflammatory reaction were noted.

At 5 and 10 days post-surgery, four rats per treatment group were euthanized by a CO_2_ overdose and skin samples were collected from each wound site of each animal. The skin samples were then divided into halves and processed for histological and gene expression analysis.

### 4.4. Histological Analysis

A total of 32 biopsies, 16 for each time point (8 for the treated and 8 for the untreated lesions), was processed for histological analysis. Skin samples were fixed in 10% neutral-buffered formalin for 24 h. Then, samples were washed and dehydrated by using a gradual dilution of ethanol; afterwards, samples were embedded in paraffin following standard procedures. After embedding, samples were cut with a microtome (Leica—RM2035, Leica Microsystems, Wetzlar, Germany) into 5 µm thick slices. For histopathological evaluation, sections were stained with hematoxylin and eosin (H&E) following the standard protocol. All sections were observed under a light microscope (Olympus Vanox AHBT3, Olympus, Tokyo, Japan). All slides were evaluated for the presence and development of granulation tissue by using a semi-quantitative score from 0 to 3 (0 absence, 1 mild, 2 moderate, and 3 abundant presence). The observations were made by one blinded operator. All data were calculated for each subject and presented as relative frequencies (%).

### 4.5. Gene Expression Analysis

A total of 32 biopsies, 16 for each time point (8 for the treated and 8 for the untreated lesions), was used for gene expression analysis by real time PCR.

Total RNA was isolated from skin samples by using TRIzol reagent (Life Technologies, Carlsbad, CA, USA). Then, the RNA extracted was assessed for its quality (260/280 nm wavelengths ratio) and quantified using a Nanodrop spectrophotometer (Thermo Scientific, Waltham, MA, USA). A total amount of 1 µg of RNA was retrotranscribed with Superscript^TM^ II Reverse Transcriptase (Invitrogen, Carlsbad, CA, USA) to obtain complementary DNA (cDNA). The cDNA was used as template for the Real Time PCR (RT-PCR) gene expression analysis using the ABI 7500 Real-Time PCR system (Applied Biosystems, Foster City, CA, USA).

In this study, the relative expression of genes involved in the wound healing process was evaluated using a specific pair of primers, which were designed using the Primer Express 3.0 software (Applied Biosystems, Foster City, CA, USA) based on the rat annotated genome sequence on the GenBank database (rat genome assembly: GCA_015227675.2) (Table 1). The efficiency of the designed primers was assessed by using the standard curve method. All pairs of primers presented an acceptable slope (between −3.8 and −3.3) with a corresponding efficiency of 90–100%. To calculate the efficiency, the ABI 7500 System SDS Software v1.5.1. (Applied Biosystems, Foster City, CA, USA) was used.

In particular, the relative expression of the following genes was evaluated: collagen 1α1 (Collagen type I, Col1α1), collagen 3α1 (Collagen type III, Col3α1), vascular endothelial growth factor A (VEGF-A), platelet-derived growth factor subunit B (PDGF-B), tumor necrosis factor α (TNF-α), transforming growth factor beta 1 (TGF-β1), interleukin-1 beta (IL-1β), interleukin-10 (IL-10), interleukin-8 (IL-8). Glyceraldehyde 3-phosphate dehydrogenase (GAPDH) and beta-actin (β-ACT) were used as reference genes to normalize the obtained data.

All experiments were run in duplicate to study the relative gene expression of each gene of interest. A melting curve analysis (dissociation curve) was performed as well to detect the non-specific amplification. The relative expression was calculated by using the 2^−ΔΔCt^ method to normalize the cDNA level of expression of the gene of interest to the reference genes. The uninjured skin was used as the calibrator sample.

### 4.6. Statistical Analysis

All data were analyzed using GraphPad Prism v9.0 software (San Diego, CA, USA). Before performing the statistical analysis, data were assessed for their Gaussian distribution by applying the Shapiro-Wilk normality test. Differences between the two groups were assessed by Student’s *t*-test with Welch’s correction. Differences presenting a *p*-value lower or equal to 0.05 were considered significant. Data were expressed as mean ± standard error of the mean (SEM).

## 5. Conclusions

Overall, our preliminary findings suggest that the MCDT might be a valuable tool in promoting and supporting wound healing: it is biocompatible as no adverse reactions were observed (neither macroscopically nor histologically), it stimulates angiogenesis and the deposition of mature collagen, and leads to an outcome comparable to the commercially available product (IDRT) chosen for this study.

## Figures and Tables

**Table 1:** Primer sequences used for the Real Time PCR.

GAPDH	FW: 5′-CCATTCTTCCACCTTTGATGCT-3′ RW: 5′-TGTTGCTGTAGCCATATTCATTGT-3′
β-ACT	FW: 5′-CCGTAAAGACCTCTATGCCA-3′ RW: 5′-AAGAAAGGGTGTAAAACGCA-3′
IL-1β	FW: 5′-GACAAGCAACGACAAAATCCC-3′ RW: 5′-TGGGTATTGTTTGGGATCCAC-3′
TGF-β1	FW: 5′-CCCCTGGAAAGGGCTCAACAC-3′ RW: 5′-TCCAACCCAGGTCCTTCCTAAAGTC-3′
TNF-α	FW: 5′-AGCCTCTTCTCATTCCTGCTC-3′ RW: 5′-GTTTGCTACGACGTGGGCTAC-3′
COL1A1	FW: 5′-ATCAGCCCAAACCCCAAGGAGA-3′ RW: 5′-CGCAGGAAGGTCAGCTGGATAG-3′
COL3A1	FW: 5′-TGATGGGATCCAATGAGGGAGA-3′ RW: 5′-GAGTCTCATGGCCTTGCGTGTTT-3′
PDGFb	FW: 5′-TGGAGTCGAGTCGGAAAGCT-3′ RW: 5′-GAAGTTGGCATTGGTGCGAT-3′
VEGF	FW: 5′-ATCATGCGGATCAAACCTCACC-3′ RW: 5′-GGTCTGCATTCACATCTGCTATGC-3′

## Data Availability

The data presented in this study are available in the article and from the authors on request.
